# Pathomechanisms of ulnar ligament lesions of the wrist in a cadaveric distal radius fracture model

**DOI:** 10.3109/17453674.2011.579517

**Published:** 2011-07-08

**Authors:** Johan H Scheer, Lars E Adolfsson

**Affiliations:** Department of Orthopaedic Surgery and Sports Medicine, Linköping University Hospital, Linköping, Sweden

## Abstract

**Background and purpose:**

Mechanisms of injury to ulnar-sided ligaments (stabilizing the distal radioulnar joint and the ulna to the carpus) associated with dorsally displaced distal radius fractures are poorly described. We investigated the injury patterns in a human cadaver fracture model.

**Methods:**

Fresh frozen human cadaver arms were used. A dorsal open-wedge osteotomy was performed in the distal radius. In 8 specimens, pressure was applied to the palm with the wrist in dorsiflexion and ulnar-sided stabilizing structures subsequently severed. Dorsal angulation was measured on digitized radiographs. In 8 other specimens, the triangular fibrocartilage complex (TFCC) was forced into rupture by axially loading the forearm with the wrist in dorsiflexion. The ulnar side was dissected and injuries were recorded.

**Results:**

Intact ulnar soft tissues limited the dorsal angulation of the distal radius fragment to a median of 32^o^ (16–34). A combination of bending and shearing of the distal radius fragment was needed to create TFCC injuries. Both palmar and dorsal injuries were observed simultaneously in 6 of 8 specimens.

**Interpretation:**

A TFCC injury can be expected when dorsal angulation of a distal radius fracture exceeds 32^o^. The extensor carpi ulnaris subsheath may be a functionally integral part of the TFCC. Both dorsal and palmar structures can tear simultaneously. These findings may have implications for reconstruction of ulnar sided soft tissue injuries.

A complex of ligaments on the ulnar side of the wrist supports the stability of the ulnocarpal and the distal radioulnar (DRU) joints. Included in this are the extensor carpi ulnaris (ECU) subsheath and the triangular fibrocartilage complex (TFCC), which is further subdivided into the radioulnar ligaments (RULs), the ulnotriquetral ligament (UT), and the ulnolunate (UL) ligament (Garcia-Elias 1998, Berger 2001). Injuries to the TFCC are common in dorsally angulated fractures of the distal radius fracture (Colle's fracture) and may adversely affect functional outcome (Lindau et al. 2000). The pathomechanics of these injuries are poorly studied, however.

During wrist arthroscopy, we have observed two lesions that are often present when treating TFCC lesions associated with distal radius fractures: (1) a separation of the floor of the ECU tendon sheath from the TFCC, and (2) an injury to the foveal insertion of the TFCC into the ulna. It seems probable that there must be a limit to how much the distal radius fragment can be displaced without rupture of the TFCC or fracture of the ulna.

We investigated the characteristics of a TFCC injury in a cadaveric fracture model of dorsally displaced fractures. We hypothesized that (1) a TFCC lesion can be expected at a certain degree of displacement and that (2) a rupture of the foveal insertion would begin in the palmar capsule and progress dorsally, due to the dorsal displacement of the distal radius fragment.

## Material and methods

16 fresh frozen cadaver extremities from donors, obtained through a non-profitable foundation, were used. They had all been transected at the mid-humerus. The median age of the donors was 53 (42–72) years. 8 were from females and 8 were from males. There was no radiographic evidence of previous distal radius or ulnar styloid fracture, or of DRU joint osteoarthritis, in any of the specimens. A radioulnar stress test (Lindau et al. 2000, Kim and Park 2008) and an ulnocarpal stability test in neutral position were performed in order to rule out pathological laxity on the ulnar side of the wrist.

### Experiment 1

For the first part, 8 specimens were used. Median palmar angulation of the joint surface, measured on a lateral view with the long axis of the radius as a reference, was 8^o^ (6–11). Through a 6-cm dorsal incision centered over Lister's tubercle, the distal radius was exposed. 2 self-drilling 3.0-mm threaded Apex pins were drilled parallel to each other, spaced 50 mm from one another, into the radius from dorsal to palmar with the help of a custom-made drill guide. The most distal pin was placed first, just proximal to the joint surface at an angle of approximately 90^o^ to the long axis of the radius. With an oscillating saw, a 50^o^ dorsal wedge osteotomy was created starting 5 mm proximal to the DRU joint and between the Apex pins, with the most distal cut parallel to the pins. The palmar cortex was sawed through, taking care to leave the palmar soft tissues intact. With the forearm in neutral rotation and the elbow resting on a digital scale, the hand of the specimen was dorsiflexed 90^o^. Pressure was applied with the palm of the investigator's hand against the palm of the specimen's hand. Care was taken only to hinge the distal fragment on the palmar cortex, to avoid dorsal displacement. A lateral radiograph was obtained while maintaining pressure. A separate observer recorded the reading on the scale when the radiograph was taken.

The TFCC was then approached from a palmar lazy-S incision ulnar to the flexor carpi ulnaris (FCU) tendon. The retinaculum was divided longitudinally to expose the TFCC and palmar capsule. The soft tissues were then severed stepwise in the following order. (1) Firstly, the floor of the ECU tendon sheath was sharply dissected off its ulnar groove and separated sharply from the dorsal radioulnar ligament. The same amount of manual pressure was re-applied with palm against palm and a second lateral radiograph was taken. (2) Secondly, the palmar DRU joint capsule was transversely incised over the ulnar head and the foveal insertions of the TFCC were sharply dissected from it. Pressure was then applied again and a third radiograph in lateral view was taken. Readings on the scale were recorded in each step.

Dorsal angulation was measured on the Apex pins on each of the digitized lateral radiographs, with zero degrees signifying the anatomical position.

Statistical analysis of dorsal angulation was done using the Mann-Whitney U test for pairs.

### Experiment 2

**Figure 1.**
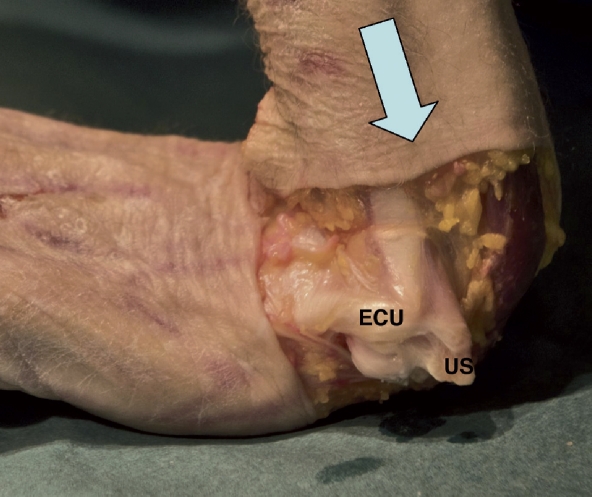
The TFCC is forced into rupture (specimen 4). The ulnar head is bald. Note the ECU tendon sheath torn out of its groove in the distal ulna. US: ulnar styloid with a type-1 fracture; bold arrow: longitudinal force applied to the forearm.

**Figure 2.**
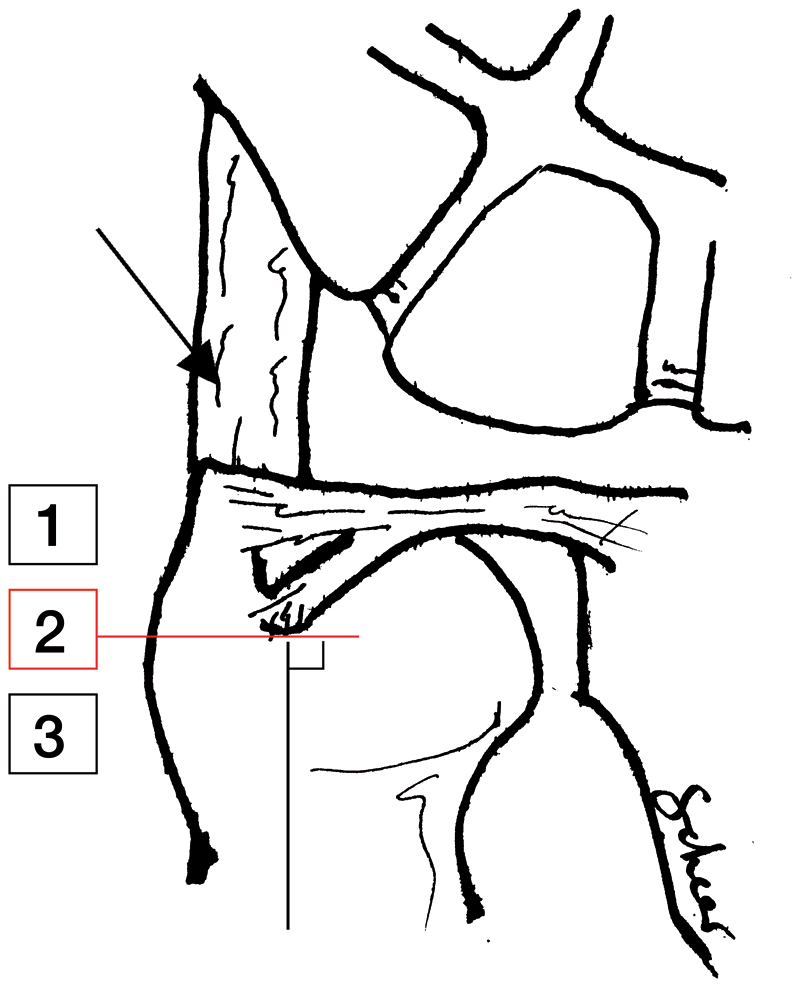
Classification of ulnar styloid fractures. Palmar view. Type 1: distal to the base where superficial horizontal fibers of the TFCC insert, as well as the dorsal UT ligament (Sasao et al. 2003). Type 2: base fracture; goes through a line perpendicular to the ulnar shaft and into the proximal limitation of the fovea, but does not involve the articular surface of the ulnar head. Type 3: proximal to a Type 2 fracture. Arrow: ECU tendon sheath.

For the next part, the other 8 specimens were used. In a similar manner as described above, a dorsally based wedge of bone was taken out (median angle 25^o^ (22–30)). The skin and retinaculum was then dissected off the ulnar side of the wrist from the extensor digiti quinti (EDQ) to the FCU tendon, leaving the ECU tendon sheath intact. The wrist was then positioned in dorsiflexion and the forearm in neutral rotation on a table and axial pressure was applied along the forearm (Figure 1). The pressure was gradually increased until the soft tissues on the ulnar side audibly or visibly ruptured. The ligaments on the ulnar side were then dissected and injuries recorded. Ulnar styloid fractures were classified as outlined in Figure 2.

## Results

### Experiment 1

The pressure applied when taking the radiographs varied between 45 N and 55 N. Severing of the ulnar soft tissues stepwise in the cadaver specimens allowed for more and more dorsal angulation in the osteotomy. With the ulnar soft tissues intact, the distal fragment could be angulated a median of 32^o^ (16–34) from the anatomical position. After sharply dissecting the ECU tendon sheath from its ulnar groove and from its attachments to the dorsal radioulnar ligaments, dorsal angulation increased by a median of 8^o^ (p = 0.02)—or in absolute values, to a median of 40^o^ (31–43). The complete severing of all ulnar insertions of the TFCC enabled the distal fragment to displace the whole width of the osteotomy, an additional median angle of 9^o^ (p = 0.01).

### Experiment 2

**Figure 3.**
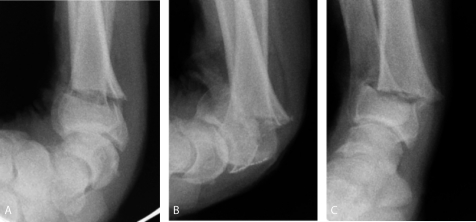
Sequence of displacement of the distal radius fragment in specimen 4. A. Before TFCC disruption. B. Maximum displacement. C. Pressure released.

When attempting to force the TFCC into rupture, nothing happened just by closing the osteotomy gap and leaving the palmar cortex in continuity. It was first when dorsal shearing of the distal fragment occurred that the restraining soft tissues yielded. When the pressure was released, the remaining soft tissues reduced the fracture to a less displaced position (Figure 3).

**Figure 4.**
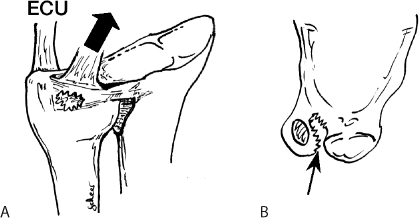
Type-1 foveal TFCC fiber disruption. A. Oblique palmar view. A hole is visible in the palmar capsule and foveal fibers are disrupted. Bold arrow: tension of the soft tissues in the direction of the UC, UT, and UL ligaments. B. Transverse view. Small arrow: the dorsal separation between the ECU tendon sheath and the TFCC.

**Figure 5.**
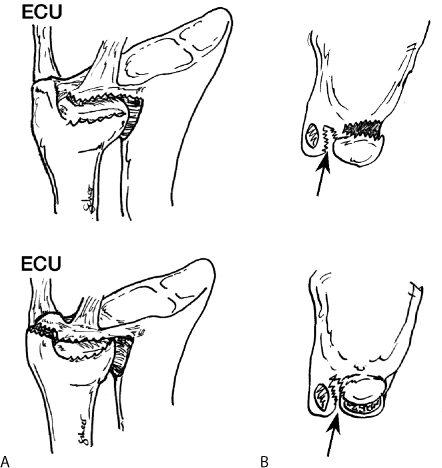
Type-2 foveal TFCC fibre disruption. A. Oblique palmar view. Complete disruption of the palmar capsule with either a sagittal rupture of the superficial fibers of the radio­ulnar ligaments (upper panel) or an ulnar styloid fracture of type 1 (lower panel). B. Transverse view. Arrow: the dorsal separation between the ECU tendon sheath and the TFCC.

**Figure 6.**
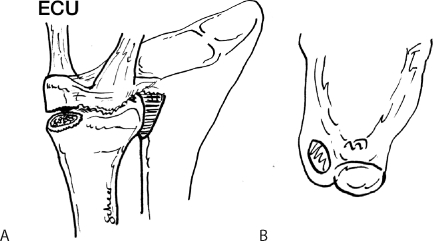
Fracture through the base of the ulnar styloid (type 2). A. Oblique palmar view. The TFCC was displaced together with the ulnar styloid fragment. B. Transverse view. Note the absence of dorsal separation between the ECU tendon sheath and the TFCC.

The injuries to the ulnar soft tissues are outlined in Figures 4–6. 6 of 8 specimens showed a combination of a dorsal injury and a palmar injury. These either had no ulnar styloid fracture (3/6) or had an ulnar styloid fracture of type 1 (3/6). The tissues that were avulsed from the tip of the ulnar styloid contained both superficial horizontal radioulnar fibers and fibers running longitudinally to the dorso-ulnar aspect of the triquetrum. Separated from this was the floor of the ECU tendon sheath, which was also torn out of its ulnar groove (Figure 1). All of this is referred to as the dorsal injury. In specimen 8, we could with certainty confirm that the dorsal injury preceded the palmar one.

Foveal fiber rupture appeared to start on the palmar side and progress dorsally. Specimen 1 had only a small palmar rupture at the palmar junction between the ulnar styloid and the ulnar head (Figure 4). Some of the palmar foveal fibers had ruptured, but most foveal attachments were intact. In the remaining 5, all palmar ligaments as well as most of the foveal fibers were avulsed, but some fibers of the dorsal radioulnar ligament remained attached to the fovea (Figure 5).

The pattern was different in the 2 specimens with an ulnar styloid fracture of type 2. Here, the base fracture audibly preceded the palmar capsule rupture and this could be confirmed on radiographs; no dorsal injuries were seen. Instead, the intact TFCC complex was displaced together with the ulnar styloid (Figure 6).

None of the specimens showed ruptures of the TFCC insertions into the radius (Palmer 1D) or ruptures of the ulnolunate or ulnotriquetral ligaments (Palmer 1C) (Palmer 1989).

## Discussion

### Experiment 1

The TFCC as a restraint for fracture displacement has been noted before in cadavers, but this has not been quantified (af Ekenstam and Hagert 1985, Pogue et al. 1990). Our findings indicate that in a patient with a distal radius fracture, one can presume an ulnar-sided ligament injury around the TFCC when the initial lateral radiograph in neutral rotation shows a dorsal angulation of more then 32^o^ over the normal palmar angle or when a dorsal angulation of 20–30^o^ is combined with a dorsal displacement of the fragment (as suggested in experiment 2). Displacement at the moment of injury might probably have been even greater (Figure 3).

In this model, we used a simple digital scale and the accuracy of measurement of the force applied may therefore have been imprecise. The angle was only measured on one radiograph; a mean of multiple readings might have added more precision. However, the calculated median of the maximum dorsal displacement of 32^o^ is just shy of the maximum of 34^o^ recorded in one of the specimens, indicating that these factors are of less importance and that this may therefore represent a fair estimation of an upper threshold angle, which may of course vary between individuals.

The point is also that the only restraining structures that changed between the different steps of the experiment were the ones sectioned. Thus, when a distal radius fragment becomes dorsally angulated, there is a pull both on the connection between the ECU subsheath and the dorsal RUL and on the TFCC.

It is of interest that by separating the ECU tendon subsheath from the dorsal radioulnar ligaments, it was possible to angulate the distal radius fragment dorsally substantially more, despite the fact that the foveal fibers were intact. This is supported by the dorsal injuries found in the forced injury model (experiment 2). It is probable that this is due to the ECU subsheath normally acting as a restraint towards displacement of the ulnar carpus relative to the ulna.

### Experiment 2

When sustaining an extraarticular fracture of the distal radius, bending, compressing, shearing, and also rotational forces may contribute to the pattern of injury (Fernandez and Jupiter 2002). To our knowledge, there have been no studies using the same method to traumatize the TFCC. Previous cadaveric studies have mainly focused on rotational forces or DRU joint distraction as the cause of TFCC injuries (Heiple et al. 1962, Adams et al. 1996). Our model, with palmar pressure and a combination of shearing and bending to cause TFCC failure, may more closely represent the pathomechanics of injuries sustained in vivo. Different patterns of injury may, however, be seen when force is applied in other directions. Also, the number of specimens in our study was small and generalization must therefore be done with caution.

We observed both dorsal and palmar injuries when forcing the TFCC into failure. It has been suggested, using a computer model, that excessive traction in the extrinsic ulnocapitate (UC) in an extended wrist could be responsible for TFCC foveal tears by pulling it out of the fovea with the hand in dorsiflexion and radial deviation (Moritomo et al. 2008). This is based on the supposed origin of the UC ligament in the fovea. This is not unequivocal, however, since some depict the insertion of this ligament onto both the ulnar styloid and the palmar radioulnar ligament (Berger 2010). Another possible mechanism may be traction of the palmar ulnotriquetral (UT) and ulnolunate (UL) ligaments, which insert in the palmar radioulnar ligament of the TFCC (Garcia-Elias 1998, Berger 2001) or at the palmar border of the fovea (Sasao et al. 2003).

The dorsal injuries we observed were a combination of a separation of the ECU subsheath from the rest of the TFCC and an avulsion of the soft tissues inserting at the tip of the ulnar styloid with or without a styloid fracture. The subsheath rupture may be a result of a simple bowstring phenomenon (Tang et al. 1998) and can be seen in Figure 1. The injury at the styloid tip may be mediated by a structure called the dorsal ulnotriquetral (dUT) ligament. This structure originates at the tip of the ulnar styloid, runs palmarly of the ECU subsheath, but is of histologically different tissue and inserts into the dorsal side of the triquetrum (Sasao et al. 2003). The similar directions but different insertions of the dUT and the ECU subsheath may explain both the coexistence of the lesions and the separation of the ECU subsheath from the horizontal parts of the TFCC, both superficial and deep portions. In the light of our findings, the ECU subsheath can be considered a functionally integral part of the TFCC, providing ulnocarpal stability.

We found another distribution of soft tissue lesions in connection with an ulnar styloid fracture of type 2. In these, the ECU subsheath and the TFCC were not separated and remained attached to the ulnar styloid. Displacement of the styloid also displaced the soft tissues en bloc. In these lesions, a combination of dorsal and palmar traction may cause the fracture by bending it off. We cannot explain the differences in distribution of injury between the specimens, but although we tried to ascertain a standardized procedure they may be attributed to anatomical variations or minor differences in displacement forces.

Despite severe displacement of the distal fragment in a distal radius fracture, frank dislocations of the DRU joint are rare. We could not induce a dislocation of the DRU joint in any of the specimens because a portion of the dorsal radioulnar ligament remained attached to the ulna. It appears likely that this is due to the direction and magnitude of the displacing force. We can only state that dorsal angulation and sheering in neutral position is unlikely to produce a DRU joint dislocation.

The most commonly used classification of TFCC injuries is the Palmer classification (Palmer 1989). This classification does not entirely cover the palmar injuries or the dorsal lesions observed here. The latter have, however, been described previously in clinical practice (Melone and Nathan 1992, Adolfsson 1994, Corso et al. 1997). If Palmer's classification is to be used as a guideline for treatment, it appears that class 1B should be redefined to include these lesions.

We believe that our study aids in understanding of the soft tissue injuries that can be associated with dorsally displaced distal radius fractures. This may be of importance, since it appears likely that some of these lesions may predispose to ulnar-sided wrist instability. Structures stabilizing the DRU as well as the ulnocarpal joint were engaged and we believe that an understanding of the possible soft tissue injury distribution is important if surgical reconstruction is considered. In addition to reinsertion of foveal fibers, repair of all injured structures would also include dorsal and palmar structures, but with the prerequisite that the osseous congruity of the DRU joint is restored.
